# Which of Our Modeling Predictions Are Robust?

**DOI:** 10.1371/journal.pcbi.1002593

**Published:** 2012-07-26

**Authors:** Rob J. De Boer

**Affiliations:** Theoretical Biology & Bioinformatics, Utrecht University, Utrecht, the Netherlands; Emory University, United States of America

## Abstract

In theoretical ecology it is well known that the steady state expressions of the variables in a food chain crucially depend on the parity of the length of the chain. This poses a major problem for modeling real food webs because it is difficult to establish their true number of trophic levels, with sometimes rare predators and often rampant pathogens. Similar problems arise in the modeling of chronic viral infections. We review examples where seemingly general interpretations strongly depend on the number of levels in a model, and on its specific equations. This Perspective aims to open the discussion on this problem.

Patients chronically infected with HIV-1 differ several orders of magnitude in the total amount of virus circulating in their blood. Individual patients approach their particular “set-point” viral load on a time scale of months, after which it remains fairly stable over a period of years. The viral set-point is a quasi steady state in which productively infected cells have a half-life of about 1 d [1]–[3] and are continuously replaced by newly infected target cells. The biological mechanism underlying the huge heterogeneity in set-points in HIV-1-infected patients is not well understood. Because genetic differences in hosts [4], [5] and viruses [6]–[8] play a role, every HIV-1-infected patient comes with its own set of parameters. One major heterogeneity in the hosts is the polymorphism in the HLA molecules activating 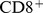 cytotoxic T lymphocytes (CTL) [5].

Fitting mathematical models to experimental data has identified several crucial parameters of this viral infection [2], and this is one of the most productive areas of mathematical biology, involving intensive collaborations between modelers, immunologists, and virologists. Several mathematical modeling studies have addressed the question of the variation in set-point viral loads [3], [9], [10]. Paradoxically, the outcome of these studies depends strongly on the design of model, and especially on the number of levels of interaction incorporated in the model [9]. Similar problems have been described in theoretical ecology, where the parity of the number of trophic levels in a model is known to influence the predicted outcome [11], [12]. Since good mathematical models are natural simplified caricatures of complex biological systems, one would hope that the predictions and interpretations inferred by analyzing these models were more robust and relatively independent of their precise set of equations.

## Model Predictions Are Not General

Let us illustrate the absence of robustness by presenting simple models for chronic viral infections, involving target cells (*T*), infected cells (*I*) producing virus (*V*), and implicitly or explicitly, an immune response (*E*). The nature of these models is basically an ecological food-chain of prey (*T*), predator (*I*), and top-predator (*E*). A first model would read:
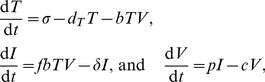
(1)where *σ* is a production term of target cells (cells d^−1^), *d_T_* the death rate of target cells (d^−1^), *b* the infection rate, 0≤*f*≤1 the fraction of successful infections, *δ* the death rate of productively infected cells (d^−1^), *p* the number of virions produced per infected cell d^−1^, and *c* the clearance rate of viral particles. The cellular immune response is implicit in this model and could affect *f*; for example, *f* = (1+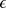 *E*)^−1^, and/or *δ*; for example, *δ* = *d_I_*+*kE*, where *E* is the magnitude of the immune response, 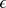 *scales their* scales their “early” effect [3], [9], [13], and *k* is a mass-action killing rate. Since the dynamics of viral particles is much faster than that of the cells [2], one typically replaces d*V*/d*t* by its quasi steady state *V* = (*p/c*)*I* to arrive at 
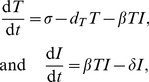
(2)where *β* = *bp*/*c*, and we have set *f* = 1 for reasons of simplicity (see below). The parameter *δ* has been estimated in hundreds of patients, varies around *δ* = 1 d^−1^, and is not correlated with the set-point viral load [3]. The most important target cell for HIV-1 is an activated CD4^+^ T cell, and this model has a fixed production term of *σ* target cells d^−1^ (which can also be modeled with a logistic growth term). During the first weeks of infection the viral load grows exponentially at a rate of approximately 1.5 d^−1^ [14]. Since *δ*≃1, one could argue that *βT*(0)≃2.5 d^−1^, where T(0) = *σ*/*d_T_* is the target cell density in the absence of infection.

Bonhoeffer et al. [3] have generalized the steady state of Equation 2 by writing a very generic model, d*T*/d*t* = *P*−*B* = 0, where *P* and *B* stand for “net production” and “infection” of target cells, respectively, and d*I*/d*t* = *B*−*δI* = 0. Using that *P* = *B*, they arrive at 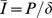, where they also let δ implicitly take the immune response into account. Because *δ* hardly varies among patients, it was argued that variation in the net production of target cells, *P*, should be responsible for the large variation observed in the set-point of the infected cells [3]. Indeed, in the presence of infection the steady state of Equation 2 can be written as:
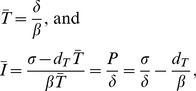
(3)Where *P* = σ−*d_T_T*, and 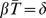 by the steady state of d*I*/d*t* in Equation 2. Thus, this model is a special case of the seemingly very general conclusion of Bonhoeffer et al. [3] that variation in *Ī* is largely due to variation in net target cell production, *P*, given that *δ* is fairly invariant.

## Adding an Explicit Immune Response

Nowak and Bangham [15] extended Equation 2 with a very simple immune response, and wrote that:
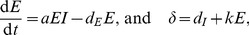
(4)where *a* is an activation rate allowing *E* to proliferate, *d_E_* and *d_I_* are normal turnover rates (d^−1^), and *k* is a mass-action killing rate. Disturbingly, if Equation 2 is extended with Equation 4, the steady state of the infected cells, *Ī*, can only be solved from Equation 4 and becomes *Ī* = *d_E_*/*a*. Since we do not expect much variation in the life spans of CTL among patients, most of the variation in *Ī* should then be due to the activation rate *a*. This seems to contradict the generic *Ī* = *P*/*δ* result derived above. However, it can be shown from the steady state of the full model that mathematically both results are in agreement (as they should be). Solving the steady state of Equation 2 and Equation 4 yields:
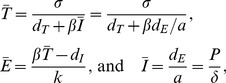
(5)where the latter is true because 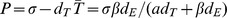 and 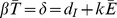.

Although it is obviously still correct that *Ī* = *P*/*δ* in the steady state of the extended model, it is no longer true that patients with a different parameter for the production of target cells—for example, different *σ*—will have a different set-point levels, *Ī*. Only differences in the activation parameter *a* (or possibly *d_E_*) can account for that. Thus, the two immune response parameters fully determine the set-point, *Ī = d_E_/a, and then the steady state of the whole model also obeys Ī = P/δ because the steady state expressions 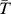 and 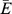 let P = δĪ. Extending the general model with an explicit immune response thus has a major effect on the predicted outcome of heterogeneity in hosts in their parameters σ and a.*

Can such a dominant role of a cytotoxic immune response be in agreement with the minor variation observed in the death rate *δ*? Yes, Nowak and Bangham [15] showed that the magnitude of the steady state immune response, 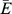, varies much less than the viral set-point. Indeed, for high activation rates, *a*>*βd_E_*/*d_T_*, the target cells in Equation 5 approach their uninfected steady state, 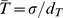, and hence the immune response approaches its maximal value 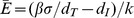, while the set-point *Ī* remains inversely related to *a* in this domain.

## More Realistic Immune Responses

**Figure 1:**
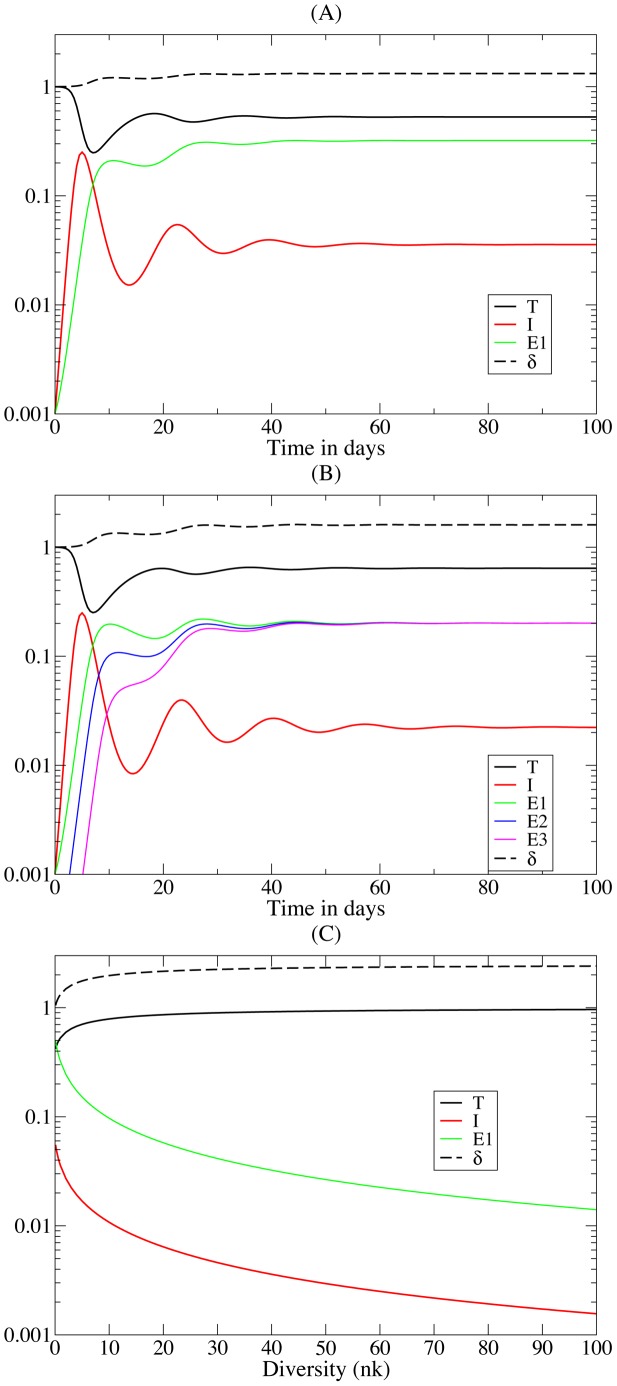
Numerical simulation of Equation 6. Without loss of generality one can scale the number of target cells in the uninfected case to *T*(0) = *σ*/*d_T_* = 1. Allowing for an initial growth rate of the infection of approximately 1.5 d^−1^ [14], we set *β* = 2.5 d^−1^ because *δ* = 1 d^−1^. Since the activated CD4^+^ T cells comprise the majority of all target cells [2], we let them to be relatively short-lived and set *d_T_* = 0.1 d^−1^. CD8^+^ effector cells should also be short-lived and we set *d_E_* = 0.1 d^−1^. Assuming that CD8^+^ T cells are activated at low doses of antigen the saturation constant *h* can be set to zero. The maximal proliferation rate of CD8^+^ T cells is approximately *p_E_* = 1 d^−1^, and we set the avidity *a_i_* = 1 for each *E_i_*. The killing rates of CTL are not known and we begin with setting *k* = 1. The initial condition in Panel (A) is *T*(0) = 1, *I*(0) = *E_1_*(0) = 10^−3^, and in Panel (B) we add two more immune responses by setting *E_2_*(0) = 10^−4^ and *E_3_*(0) = 10^−5^. The model behavior is somewhat too oscillatory, but it is known that this can be repaired by allowing for two stages of the infected cells [20]. The effect of the diversity, *n*, of the immune response on the steady state can be investigated more generally by making all immune responses equal (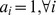), and observing that 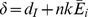, where all 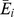 are the same. The dependence of steady state and *δ* on the diversity, *n*, is depicted in Panel (C) by plotting the steady state as a function of *nk*, where the line marked by *E_i_* depicts the size of a single immune response. We observe that 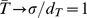, and that *δ*→2.5 d^−1^, for *nk*>10. For higher killing rates this happens earlier—that is, for *k* = 10 going from *n* = 1 to *n* = 10 immune response makes hardly any difference in the total killing rate *δ*. For *k* = 1 this axis would obviously correspond to the diversity, and then the total immune response, *nE_i_*, increases with *n* (not shown).

Equation 4 for the immune response is very simple, and yet different results will be obtained if one were to take the equally simple d*E*/d*t* = *aI*−*d_E_E*, which assumes that the total production of effectors is limited by *I* [9]. Another problem with Equation 4 is that one cannot model several immune responses that are together controlling a chronic infection, because this model would predict competitive exclusion, allowing only the response with the largest *a* to survive. We have proposed immune response functions based upon a competitive saturation term, resembling a Beddington functional response [12], [16], which can be derived by making a special quasi steady assumption [17]–[20]: 
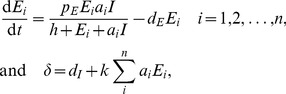
(6)where 0≤*a_i_*≤1 is the “avidity” of immune response *i* for infected cells, *p_E_* is the maximum division rate (d^−1^), *h* is a saturation constant (that can be set to *h* = 0 because during a chronic infection typically *E_i_*+*a_i_I*≫*h*), and we allow for *n* immune responses. The steady state of Equation 6 is 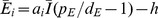, saying that the magnitude of each immune response is approximately proportional to the set-point *Ī* weighted by the avidity. For one immune response, the steady state of the full model is obtained by solving a quadratic equation, and selecting the positive root knowing that *p_E_*>*d_E_*, we have 
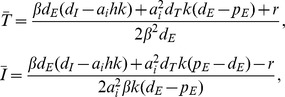
where
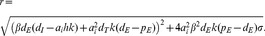
(7)Importantly the steady state expression for *Ī* now contains all parameters of the model, and one can show numerically that increasing *σ* increases the set-point and that an increase of *a* or *k* decreases the set-point (see Figure 1C and compare the related model of Müller et al. [9]). Thus, rather than having a single parameter determining *Ī*, parameters now have a more pleiotropic effect that seems much better in agreement with the fact that several factors influence the viral set point [4]–[8].

This model has similar saturation effects as the parameter *a* in Equation 5. For instance, if the collective immune response is strong, because *k* or *n* is large, the target cells will again approach their healthy steady state, 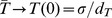 (see Figure 1C). Indeed one can compute a maximal steady state immune response by arguing that in 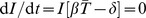 the maximal “replication rate” of infected cells, 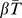, is obtained when 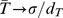. This implies that at steady state *δ_max_* = *βσ*/*d_T_*≃2.5 d^−1^. Importantly, when *δ* is defined by Equation 6, this means that increasing the diversity of the immune response, *n*, is not expected to proportionally increase *δ* (Van Deutekom, Wijnker, & De Boer, unpublished) (even though we have no direct competition between the immune responses [21]). Instead, the more immune responses there are, the smaller the contribution of each immune response to *δ*, ultimately approaching (*βσ*/*d_T_*–*d_I_*)/*n*, which is confirmed numerically in Figure 1. This could help to explain why there is so little variation in *δ* among patients, because *δ* would tend to reflect the maximal growth rate of the virus. Similar arguments apply when one reconsiders the early immune response in 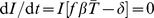, where 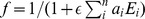 and *δ* = *d_I_*, delivering 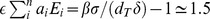 when 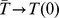 and *δ* 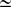
1. Both results suggest that the virus will evolve only few immune escapes when it is controlled by a diverse immune response, since escaping from each of them provides little advantage [21].

Because results have changed with every extension of the model, we should ask ourselves how generic this latter result is. Indeed, one could extend Equation 6 with exhaustion of CD8^+^ T cells during chronic infections [22] and/or add a level of regulatory T cells that down-regulate T cell responses. However, the result is obtained from setting d*I*/d*t* = 0 only, and observing that 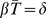, and does not require other steady states besides the intuitive argument that 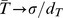 when k→∞ (see Figure 1C). Thus, whenever the diversity of the immune response is hardly changing the density of target cells, 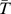, the killing per CTL response should be inversely related to the diversity, *n*.

## Parameters That Vary Should Be Functions

Müller et al. [9] have discussed similar problems with explaining the variation in HIV-1 set-points, and proposed that given the non-robustness of steady state expressions, it is better to estimate parameter values from measured steady state values. Demonstrating that such values are robust to changes in the model structure, they proceeded by arguing that parameters that vary widely between patients are probably more complicated processes—that is, functions depending on other variables—and not extremely heterogeneous constants. An excellent example is the large variation in *a* that the model of Equation 4 required to explain the large heterogeneity in viral set-points. Replacing the parameter *a* by a saturation function like that of Equation 6 allows one to explain the heterogeneity in viral-loads by combinations of small variations in several parameters [9].

## Non-Cytolytic and/or Early Killing

An important issue in the cellular immune response to HIV-1 is that the response involves more than the mere killing of productively infected cells. There may be cytolytic, and/or non-cytolytic, responses by CD8^+^ T cells before the infected cells actually start to produce virus (i.e., during their eclipse phase). This has been addressed by setting *f*<1 in Equation 1 [9] and by allowing for two sub-populations of infected cells [13]. In contrast to the models discussed above [23], these extended models are in good agreement with recent experiments where the chronic steady state is perturbed by elimination the CD8^+^ T cells [9], [13]. Thus, we again face a situation calling for improving the model by changing its equations.

## Discussion

It seems a hidden assumption in mathematical biology that generic mathematical results tend to carry over to similar other models. Disturbingly, the identification of the crucial parameters determining the set-point viral load levels have changed with every reasonable model extension discussed above. The precise reason for this absence of robustness remains unclear.

One particular problem comes about when steady state results from other equations are substituted into to the equation of interest, as illustrated above by the change of interpretation when the model of Equation 2 was extended with Equation 4. This approach of substituting terms will be most precarious when the actual number of levels implemented in a model determines its steady state expressions [11], [12]. The problem seems to most prevalent in Lotka-Volterra type models, in which in several equations *x* can be cancelled from the d*x*/d*t* equation, implying that 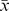 has to be solved from another level. Equation 6 is a counterexample because *E_i_* cannot be cancelled from d*E_i_*/d*t* due to density-dependent effects within the *E_i_* population, and indeed in that model the set-point became dependent on almost all parameters of the model [9]. Thus, predictions that do not rely on substituting steady state results seem to be more reliable than those that do require terms from other equations.

Arguing along these lines, the famous estimation of the death rate, *δ*, of virally infected cells from the downslope of the viral load in patients started on effective treatment [1], [24] seems a good example of a robust interpretation because one basically simplifies Equation 1 into a model like d*I*/d*t* = −*δI* and d*V*/d*t* = *pI*−*cV* by assuming *b*→0 to implement an effective treatment blocking *de novo* infections. Nevertheless, there remains to be discussion on how to interpret the various downslopes in the viral load observed during treatment [25]–[27] and during experiments [28], [29], and the results similarly depend on the number of compartments and the complexity of the models employed to describe the data. However, this is a different discussion relating to the more general problem of parameter identifiability in modeling data [30], [31].

Obviously, the question raised in this Perpective should not lead to the conclusion that mathematical modeling cannot capture biological complexity in a meaningful way, or that one should generally aim for models capturing much more biological detail. A large model will suffer from similar problems, but they will be much more difficult to detect. Modeling in biology helps us to “think more clearly” about complex problems [32], and helps us to better interpret quantitative experimental data in terms of the underlying biological processes. Instead, we have to open the discussion on how to treat the “boundary” problem of limiting the number of levels of regulation, and the complexity of the terms, in our simple mathematical models of complex biological systems.
